# The Effect of Lockdown and Physical Activity on Glycemic Control in Italian Children and Young Patients With Type 1 Diabetes

**DOI:** 10.3389/fendo.2021.690222

**Published:** 2021-07-13

**Authors:** Nicola Minuto, Marta Bassi, Carolina Montobbio, Francesco Vinci, Claudia Mercuri, Francesca Nastasia Perri, Mara Cabri, Maria Grazia Calevo, Giuseppe d’Annunzio, Mohamad Maghnie

**Affiliations:** ^1^ Department of Pediatrics, IRCCS Istituto Giannina Gaslini, Genova, Italy; ^2^ Department of Pediatrics, IRCCS Istituto Giannina Gaslini, University of Genova, Genova, Italy; ^3^ Department of Neuroscience, Rehabilitation, Ophtalmology, Genetics, Maternal and Child Health, University of Genova, Genova, Italy; ^4^ Epidemiology and Biostatistics Unit, IRCCS Istituto Giannina Gaslini, Genova, Italy

**Keywords:** type 1 diabetes, COVID-19, continuous glucose monitoring, children, glycemic control, physical activity

## Abstract

**Aims:**

The purpose of the study was to evaluate the impact of the lockdown established by the Italian government to limit the spread of Coronavirus disease (COVID-19) on glycemic control in a large sample of patients with type 1 diabetes (T1D) based on age, type of insulin therapy, number of telemedicine visits and physical activity.

**Material and Methods:**

We retrospectively evaluated glycemic control in young T1D patients using the DexcomG6^®^ system before the Italian lockdown (February 10–23, 2020—Time 0) and during lockdown (April 17–30, 2020—Time 1). Data on age, type of insulin therapy, number of telemedicine visits and physical activity of 202 patients with T1D and a median age of 18.2 years (range: 6–39) were collected.

**Results:**

Data showed a significant improvement of TIR from 54.58% at T0 to 59.09% at T1 (p ≤0.0001). Glycemic control improved significantly in patients ≥14 years old, showing the best outcome in the “university students and young adults” group (55.40% at T0 and 61.37% at T1, p ≤0.001). All patients reduced physical activity during lockdown; in the 56 patients of “intense physical activity” group both at T0 and T1 TIR increased from ±56.91 to 64.11% (p ≤0.0001).

**Conclusions:**

Overall, the lockdown led to an unexpected improvement in glycemic control of young patients with T1D. A healthier and stressless lifestyle changes in association with the maintenance of physical activity resulted in a significant age-proportional improvement in glycemic control.

## Introduction

At the beginning of 2020, Coronavirus rapidly spread in many areas of the world, causing great public health concerns (1, 2). Coronavirus disease (COVID-19), if associated to diabetes mellitus, is characterized by higher mortality in adult patients (3–6). However, some reports suggest that in T1D patients younger than 25 years, the infection shows less severe and fairly similar clinical characteristics to healthy children and young people of the same age, who rarely require hospitalization (7–11). The contagiousness, clinical characteristics and mortality of infections due to the new COVID variants are not yet fully understood and may be different from those reported in the studies mentioned. To reduce the spread of Coronavirus, some governments have established restrictive policies. In particular, the Italian Government has ordered the first emergency measures starting from February 23, 2020 (suspension of school, sports activities and meetings) and a national quarantine starting from March 9, 2020 (movement restricted except for necessity). These restrictions may have negatively impacted glycemic control in T1D patients by reducing physical exercise and encouraging a sedentary lifestyle (12). Furthermore, during the lockdown period, all scheduled outpatient and hospital activities were suspended except for emergencies and telemedicine became the only and powerful tool to assess glycemic control (13).

Continuous glucose monitoring (CGM) is widely recognized as an extremely useful tool to monitor and improve glycemic control in T1D patients. It is well established that parameters indicating a good metabolic control are no longer limited to the concept of HbA1c alone, but they are extended also to ”time in range” (TIR = 70–180 mg/dl), ”time below range” (TBR <70 mg/dl), and ”time above range” (TAR >180 mg/dl) (14–18).

Despite the concerns of diabetologists, some studies unexpectedly showed that glycemic control in T1D patients remained stable or improved during the lockdown period (19–34). However, these studies included both small and over-selected samples of patients, with different treatment schedules or adult patients. In contrast, one study in which non-CGM users were included showed worsening of glycemic control in children with type 1 diabetes (33).

The aim of our study was to evaluate the glycemic control in a large sample of young T1D patients during the lockdown period with particular attention to the role of age, type of insulin therapy, number of telemedicine visits and physical activity.

## Material and Methods

### Study Design and Study Population

This retrospective observational cohort study included young T1D patients followed at the G. Gaslini Hospital, Regional Diabetes Center (IRCCS Istituto Giannina Gaslini, University of Genova, Italy), aged 6–39 years old, who were using the Dexcom G6^®^ CGM system with a percentage of use> 70%. Patients with T1D diagnosed after February 10, 2019 and patients with psychiatric disorders, pregnancy and in chronic therapy with drugs that interfere with glycemic control (such as corticosteroids or chemotherapy) were excluded.

Because of the retrospective nature of the study the ethic approval and informed consent already signed by parents and/or patients at the disease onset and renewed yearly, in which they agree on the use of clinical data for research purposes, were used. In addition, all parents and patients provided a specific informed consent for the collection of data.

The study was conducted in accordance with the Declaration of Helsinki and International Conference on Harmonization Good Clinical Practice.

### Study Outcomes

The primary outcome was to compare the percentage of TIR (70–180 mg/dl), TAR (>180 mg/dl), and TBR (<70 mg/dl) in the period immediately preceding the lockdown (Italian DPCM of March 9, 2020) and during the lockdown period in young T1D patients using the Dexcom G6^®^ CGM system for glucose monitoring. The secondary endpoints were to evaluate changes in glycemic control in relation to age, type of insulin therapy (multiple daily injection—MDI or continuous subcutaneous insulin infusion—CSII), number of telemedicine visits and physical activity.

### Data Collection

We gathered data on glycemic control from the Dexcom Clarity software^®^ reports of observation in the frame time of 2 weeks. We decided to compare data of the 14 days before the lockdown (February 10–23, 2020—Time 0) and data of 14 days during the lockdown period (April 17–30, 2020—Time 1).

We identified 202 patients (53% male) who fulfilled the study eligibility criteria. The following data were collected for each patient: demographic data (age and gender), age at clinical onset of T1D, type of insulin therapy (MDI or CSII), number of telemedicine visits in the period from Time 0 (T0) to Time 1 (T1). In addition, we collected information on physical activity before and after lockdown (T0 and T1). We defined “none physical activity” as the total lack of physical activity, ”regular physical activity” as exercise time of less than 3 h per week and “intense physical activity” as exercise time of at least 3 h per week.

For the stratified data analysis, we divided patients into age groups according to the Italian career school path: “primary school children” (≥6 yrs; <10 yrs), “first grade secondary school children” (≥10 yrs; <14 yrs), “second grade secondary school students” (≥14 yrs; <18 yrs), and “university students and young adults” (≥18 yrs).

### Statistical Methods

Data are described as mean and standard deviation (SD) or median and IQR for continuous variables, and as absolute and relative frequencies for categorical variables. The normal distribution of the variables was assessed using the Kolmogorov–Smirnov test. Non-parametric analysis (Mann–Whitney U-test, Kruskal–Wallis test, Wilcoxon test) for continuous variables and the Chi square or Fisher’s exact test for categorical variables were used to measure differences between groups. Multivariate analysis was performed to analyze the association between glycemic control (glycated hemoglobin and TIR) and exercise subgroup, age, number of telemedicine visits, gender and type of insulin therapy before and during the lockdown. P values ≤0.05 were considered statistically significant, and all P values were based on two tailed tests. Statistical analysis was performed using SPSS for Windows (SPSS Inc., Chicago, Illinois USA).

## Results

The median age of the 202 participants at observation was 17 years (range: 6–39 yrs) with a mean of 18.30 ± 6.43 yrs; median age at T1D onset was 8 years (range: 1–20 yrs) with a mean of 8.10 ± 4.18 yrs; median duration of disease was 9 years (range: 1–31 yrs) with a mean of 10.20 ± 6.72 yrs. Among the 202 patients included, 168 (83.2%) used CSII and 34 (16.8%) used MDI; 112 patients (55.4%) made a single telemedicine visit, 48 (23.8%) did not perform any visit and 42 (20.8%) made two or more telemedicine visits during the lockdown period. The population studied was divided into the following age groups: 7 (3.5%) ”primary school children”, 42 (20.8%) ”first grade secondary school children”, 58 (28.7%) ”second grade secondary school students” and 95 (47.0%) ”university students and young adults” (**Table 1**). No patient contracted COVID infection during the study period.

**Table 1:** Demographic data, type of insulin therapy and weekly exercise time at T0.

	(n = 202)
**Age at evaluation** yrs, *mean ± SD*	18.30 ± 6.43
* median (range)*	17 (6–39)
**Age at T1D onset** yrs, *mean ± SD*	8.10 ± 4.18
* median (range)*	8 (1–20)
	
	**N (%)**
**Gender**	
Female N (%)	95 (47)
Male N (%)	107 (53)
**Type of insulin therapy**	
CSII N (%)	168 (83.2)
MDI N (%)	34 (16.8)
**Telemedicine visit**	
None N (%)	48 (23.8)
Single visit N (%)	112 (55.4)
2 or more visits N (%)	42 (20.8)
**School grade**	
Primary school N (%)	7 (3.5)
First grade secondary school N (%)	42 (20.8)
Second grade secondary school N (%)	58 (28.7)
University students/young adults N (%)	95 (47.0)
**Physical activity, exercise time**	
No physical activity N (%)	38 (19.6)
Physical activity <3 h per week N (%)	27 (13.9)
Intense physical activity ≥3 h wks N (%)	129 (66.5)

CSII, continuous subcutaneous insulin infusion; MDI, multiple daily injection.

Exercise time was collected at T0 for 194/202 patients, of whom 38 (19.6%) did not engage in physical activity, 27 (13.9%) engaged in regular physical activity (<3 h per week) and 129 (66.5%) engaged in intense physical activity (≥3 h per week).

The mean percentage of TIR increased from 54.58 ± 16.7 % at T0 to 59.09 ± 17.7% at T1 (p ≤0.0001), which corresponded to +4.5 percentage points; this mean difference amounted to 1.1 h per day spent in TIR (**Table 2**). The difference in the percentage of TAR (>180 mg/dl) was −4.0 percentage points (42.48 ± 17.78% at T0 *vs* 38.50 ± 18.45% at T1, p ≤0.0001), a difference that amounted to 1.0 h per day. The difference in the percentage of TAR >250 mg/dl was −3.5 percentage points (16.25 ± 12.31% at T0 *vs* 12.81 ± 11.59% at T1, p ≤0.0001), a difference that amounted to 48 min per day. The difference in the percentage of TBR (<70 mg/dl) was −0.4 percentage points (2.83 ± 2.91% at T0, 2.38 ± 3.06% at T1, p ≤0.002), a difference that amounted to 6 min per day. The difference in the percentage of TBR <54 mg/dl was −0.1 percentage points (0.59 ± 0.92% at T0, 0.48 ± 1.06% at T1, p ≤0.0001).

**Table 2:** Primary outcome: Glycemic control at T0 and T1.

	T0 (N = 202)	T1 (N = 202)	p-value
Coefficient of variation (%)	36.53 ± 5.76	34.76 ± 5.76	≤0.0001
SD (mg/dl)	64.26 ± 14.26	59.15 ± 13.78	≤0.0001
HbA1c (%)	7.76 ± 1.04	7.56 ± 1.05	≤0.0001
TIR (%)	54.58 ± 16.67	59.09 ± 17.65	≤0.0001
TAR (%)	42.48 ± 17.78	38.50 ± 18.45	≤0.0001
TAR >250 mg/dl (%)	16.25 ± 12.31	12.81 ± 11.59	≤0.0001
TBR (%)	2.83 ± 2.91	2.38 ± 3.06	≤0.002
TBR <54 mg/dl (%)	0.59 ± 0.92	0.48 ± 1.06	≤0.01
Mean glucose value (mg/dl)	176.16 ± 29.87	170.18 ± 30.14	≤0.0001
Sport (hours/week)	4.64 ± 4.24	2.46 ± 3.22	≤0.0001

SD, standard deviation; TIR, time in range (70–180 mg/dl); TAR, time above range (>180 mg/dl); TAR >250, time above range (>250 mg/dl); TBR, time below range (<70 mg/dl); TBR <54, time below range (<54 mg/dl).

Data also showed a significant decrease of mean glycemic values (176.16 ± 29.87 mg/dl at T0 and 170.18 ± 30.14 mg/dl at T1, p ≤0.0001) and estimated HbA1c (7.76%—61 mmol/mol at T0 and 7.56%—59 mmol/mol at T1, p ≤0.0001) (**Table 2**).

Multivariate analysis was performed to determine the prognostic factors affecting glycemic control (glycated hemoglobin and TIR). Age, exercise subgroup, number of telemedicine visits, gender and type of insulin therapy before and during the lockdown were not significant.

**Figure 1:**
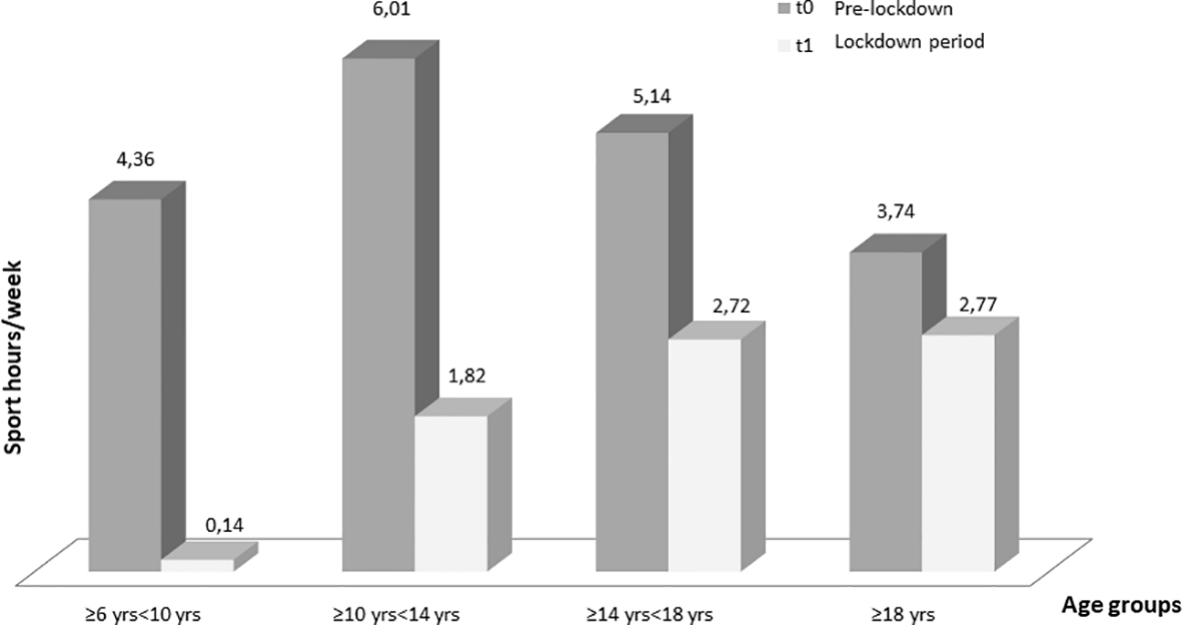
Changes in weekly sports hours for each age at T0 (before the lockdown) and at T1 (lockdown period). Only one children of seven in “primary school children” group maintained physical activity (**Table 4**).

Regarding the different age groups analyzed, glycemic control improved, although not statistically significant, in “primary school children” group (TIR 58.33 ± 21.14% at T0 and 61.49 ± 19.76% at T1) and remained almost stable in the “first grade secondary school children” group (TIR 55.74 ± 16.30% at T0 and 56.84 ± 16.91% at T1). On the other hand, glycemic control significantly improved in the “second grade secondary school students” group (51.96 ± 16.73% at T0 and 56.71 ± 19.42% at T1, p ≤0.005) and even more in the “university students and young adults” group (55.40 ± 16.53% at T0 and 61.37 ± 16.62% at T1, p ≤0.001) (**Table 3**). A statistically significant reduction in the weekly sports hours was observed in all groups: in ”primary school children” group (4.36 ± 0.94 h at T0 and 0.14 ± 0.38 h at T1, p = 0.02), in “first grade secondary school children” group (6.01 ± 4.06 h at T0 and 1.82 ± 2.32 h at T1, p ≤0.0001), in “second grade secondary school students” group (5.14 ± 4.20 h at T0 and 2.72 ± 3.40 h at T1, p ≤0.0001and in “university students and young adults” group (3.74 ± 4.33 h at T0 and 2.7 ± 3.49 h at T1, p <0.03) (**Figure 1**).

**Table 3:** Secondary outcome: Glycemic control in relation to age groups at T0 and T1.

	Age ≥6 y <10 y (N = 7)			Age ≥10 y <14 y (N = 42)			Age ≥14 y <18 y (N = 58)			Age ≥18 y (N = 95)		
	T0	T1	p-value	T0	T1	p-value	T0	T1	p-value	T0	T1	p-value
	Mean ± SD Median (IQR)	Mean ± SD Median (IQR)		Mean ± SD Median (IQR)	Mean ± SD Median (IQR)		Mean ± SD Median (IQR)	Mean ± SD Median (IQR)		Mean ± SD Median (IQR)	Mean ± SD Median (IQR)	
Coefficient of variation (%)	36.99 ± 9.19	34.07 ± 5.25	0.24	37.55 ± 6.02	34.13 ± 5.68	**≤0.0001**	36.92 ± 5.24	36.13 ± 5.34	0.31	35.82 ± 5.66	34.27 ± 6.02	**0.001**
SD (mg/dL)	61.14 ± 15.91	55.29 ± 13.34	0.07	65 ± 14.01	58.43 ± 11.74	**≤0.0001**	67.33 ± 15.52	63.40 ± 15.08	**0.002**	62.28 ± 13.27	57.16 ± 13.43	**≤0.0001**
HbA1c (%)	7.54 ± 1.08	7.33 ± 1.12	0.15	7.66 ± 1.01	7.62 ± 0.96	0.53	7.95 ± 1.09	7.76 ± 1.31	**0.03**	7.71 ± 1.02	7.42 ± 0.89	**≤0.0001**
TIR (%)	58.33 ± 21.14	61.49 ± 19.76	0.50	55.74 ± 16.30	56.84 ± 16.91	0.28	51.96 ± 16.73	56.71 ± 19.42	**0.005**	55.40 ± 16.53	61.37 ± 16.62	**≤0.0001**
TAR (%)	39.21 ± 22.25	35.27 ± 20.99	0.13	40.31 ± 16.77	40.96 ± 17.78	0.97	45.38 ± 18.01	40.84 ± 20.75	**0.02**	41.92 ± 17.79	36.22 ± 16.98	**≤0.0001**
TAR >250 mg/dl (%)	12.07 ± 11.14	9.56 ± 11.15	0.50	16.36 ± 12.86	12.74 ± 9.99	**0.01**	18.63 ± 12.13	16.22 ± 15.09	**0.004**	15.06 ± 12.18	11.01 ± 9.30	**≤0.0001**
				11.65 (16.40)	10.70 (11.85)			120 (21.60)		12.30 (16.70)	8.50 (15.70)	
TBR (%)	2.49 ± 3.09	3.24 ± 3.25	0.12	3.34 ± 3.36	2.20 ± 2.51	**0.002**	2.60 ± 2.41	2.32 ± 2.14	0.39	2.77 ± 3.0	2.42 ± 3.70	**0.04**
				2.50 (3.70)	1.40 (2.70)		1.80 (3.00)	1.60 (3.00)		1.80 (3.60)	1.30 (2.30)	
TBR <54 mg/dl (%)	0.79 ± 1.60	0.87 ± 1.32	0.35	0.80 ± 1.17	0.49 ± 0.94	**0.03**	0.52 ± 0.79	0.40 ± 0.50	0.58	0.53 ± 0.79	0.49 ± 1.32	0.06
	0.20 (0.20)	0.40 (0.60)		0.40 (0.80)	0.20 (0.70)		0.20 (0.50)	0.20 (0.60)		0.20 (0.50)	0.10 (0.40)	
Mean glucose (mg/dl)	169.7 ± 30.87	163 ± 31.34	0.09	173.43 ± 29	172 ± 27.43	0.43	181.6 ± 31.41	175.9 ± 37.64	0.02	174.6 ± 29.23	166.4 ± 25.54	**≤0.0001**
Sport (hours/week)	4.36 ± 0.94	0.14 ± 0.38	**0.02**	6.01 ± 4.06	1.82 ± 2.32	**≤0.0001**	5.14 ± 4.20	2.72 ± 3.40	**≤0.0001**	3.74 ± 4.33	2.77 ± 3.49	**0.03**
		0.00 (0.00)		5.00 (3.75)	1.00 (2.00)		4.00 (4.00)	2.00 (4.00)		3.00 (5.40)	2.00 (4.00)	

SD, standard deviation; TIR, time in range (70–180 mg/dl); TAR, time above range (>180 mg/dl); TAR >250, time above range (>250 mg/dl); TBR, time below range (<70 mg/dl); TBR <54, time below range (<54 mg/dl). bold = statistically significant values.

All patients, regardless of age group, reduced the weekly hours of physical activity during the lockdown and changed their sports habits; 86.8% of patients who did not exercise at T0 continued to be sedentary, 29.6% of patients who were engaged in regular physical activity became sedentary and 40.7% started exercising intense physical activity. In addition, 27.9% of patients who did intense physical activity became sedentary, 28.7% reduced their weekly sports hours and 43.4% maintained their habits. At T1, stratifying by age groups, there was an important reduction in physical activity in the “first and second grade secondary school students” groups (10 to 18 years old), and a slighter reduction in the “university students and young adults” group (>18 years old). Only one child out of seven in the “primary school children” group maintained physical activity (**Table 4**).

**Table 4:** Study population divided by age and by sport group at T0 and T1 (N = 194).

	T0			T1		
	No sport (N = 38)	Sport <3 h (N = 27)	Sport ≥3 h (N = 129)	No sport (N = 77)	Sport <3 h (N = 46)	Sport ≥3 h (N = 71)
	N (%)	N (%)	N (%)	N (%)	N (%)	N (%)
**Age ≥6 y <10y (N = 7)**	0	0	7 (100)	6 (85.7)	1 (14.3)	0
**Age ≥10 y <14 y (N = 40)**	2 (5)	4 (10)	34 (85)	14 (35)	18 (45)	8 (20)
**Age ≥14 y <18 y (N = 57)**	8 (14)	8 14)	41 (72)	23 (40.4)	10 (17.5)	24 (42.1)
**Age ≥18 y (N = 90)**	28 (31.1)	15 (16.7)	47 (52.2)	34 (37.8)	17 (18.9)	39 (43.3)

The pre-lockdown (T0) differences in glycemic control in relation to exercise were not statistically significant, although TIR was proportionally higher in relation to the weekly hours of physical activity: TIR 51.56 ± 16.74% in the “none activity” group, 55.51 ± 14.41% in the “regular physical activity” group and 55.63 ± 17.18% in the “intense physical activity” group; the same difference in glycemic control is observed during the lockdown: TIR 54.12 ± 17.01% in the “none activity” group, 59.69 ± 19.30% in the “regular physical activity” group and 63.98 ± 16.29% in the “intense physical activity” group. Furthermore, there is a statistically significant difference in glycemic control between the “no activity” group (n 77—TIR 54.12 ± 17.0 %), and the “intense physical activity” group (n 71—TIR 63.98 ± 16.3 %) at T1 (p value 0.0001) (**Table 5**).

**Table 5:** Secondary outcome: Glycemic control in relation to physical activity at T0 and T1 (N = 194).

	T0						T1					
	No sport (N = 38)	Sport <3 h (N = 27)	Sport ≥3 h (N = 129)	p-value No sport *vs* Sport <3 h	p-value No sport *vs* Sport ≥3 h	p-valueSport <3 h *vs* Sport ≥3 h	No sport (N = 77)	Sport <3 h (N = 46)	Sport ≥3 h (N = 71)	p-value No sport *vs* Sport <3 h	p-value No sport *vs* Sport ≥3 h	p-value Sport <3 h *vs* Sport ≥3 h
	Mean ± SD median (IQR)	Mean ± SD median (IQR)	Mean ± SD median (IQR)				Mean ± SD median (IQR)	Mean ± SD median (IQR)	Mean ± SD median (IQR)			
Coefficient of variation (%)	36.47 ± 5.88	34.48 ± 5.99	36.92 ± 5.73	0.17	0.68	**0.04**	35.39 ± 5.47	33.06 ± 5.91	35.09 ± 6.04	0.02	0.58	0.09
SD (mg/dl)	66.71 ± 15.53	61.07 ± 15.04	63.96 ± 13.97	0.19	0.40	0.28	63.04 ± 14.18	55.72 ± 12.94	57.14 ± 13.47	**0.01**	**0.02**	0.78
HbA1c (%)	8.02 ± 1.13	7.77 ± 0.87	7.67 ± 1.05	0.56	0.15	0.49	7.85 ± 1.11	7.53 ± 1.12	7.28 ± 0.89	0.11	**0.002**	0.26
TIR (%)	51.56 ± 16.74	55.51 ± 14.41	55.63 ± 17.18	0.42	0.25	0.86	54.12 ± 17.01	59.69 ± 19.30	63.98 ± 16.29	0.07	**0.0001**	0.28
TAR (%)	49.90 ± 18.13	42.83 ± 15.38	41.07 ± 18.23	0.63	0.21	0.56	44.57 ± 18.04	38.32 ± 20.42	33.24 ± 16.52	0.09	**0.001**	0.19
TAR >250 mg/dl (%)	18.94 ± 14.45	13.97 ± 10.04	15.78 ± 12.19	0.25	0.30	0.64	16.07 ± 13.09	12.16 ± 12.16	9.94 ± 8.91	**0.05**	**0.002**	0.63
	15.25 (22.60)	11.40 (16.10)	12.30 (17.80)				13.50 (18.50)	8.55 (17.30)	8.70 (10.60)			
TBR (%)	2.71 ± 3.13	1.53 ± 2.14	3.11 ± 2.96	0.07	0.25	**0.001**	2.32 ± 2.64	2.0 ± 2.24	2.66 ± 3.95	0.53	0.70	0.29
	2.05 (3.70)	0.70 (1.30)	2.10 (3.50)				1.30 (2.80)	1.20 (3.10)	1.60 (2.80)			
TBR <54 mg/dl (%)	0.41 ± 0.59	0.41 ± 1.01	0.67 ± 0.98	0.06	0.29	**0.003**	0.51 ± 0.96	0.29 ± 0.41	0.58 ± 1.44	0.23	0.62	0.48
	0.20 (0.40)	0.10 (0.30)	0.30 (0.70)				0.20 (0.50)	0.10 (0.40)	0.10 (0.50)			
Mean glucose (mg/dl)	183.47 ± 33.66	176.19 ± 24.79	173.59 ± 30.18	0.50	0.15	0.51	178.45 ± 31.89	169.3 ± 32	162.3 ± 25.45	0.11	**0.002**	0.30

SD, standard deviation; TIR, time in range* (70–180 mg/dl); TAR, time above range (>180 mg/dl); TAR >250, time above range (>250 mg/dl); TBR, time below range (<70 mg/dl); TBR <54, time below range (<54 mg/dl). bold = statistically significant values.

We analyzed the data relating to the 129 patients belonging to the “intense physical activity” group at T0; we observed that the 56 subjects included in the “intense physical activity” group both at T0 and at T1 showed a statistically significant improvement in glycemic control in the lockdown period compared to the “none activity” group (54.76 ± 16.04% *vs* 64.11 ± 16.29%, p = 0.006) (**Table 6**). Moreover, the mean percentage of TIR increased from 56.91 ± 17.13% at T0 to 64.11 ± 16.29% at T1 (p ≤0.0001).

**Table 6:** Analysis of the data at T1 of the 129 patients belonging to the “intense physical activity” group at T0.

	T1(N = 129)					
	No sport (N = 36)	Sport <3 h (N = 37)	Sport ≥3 h (N = 56)	p-value no sport *vs* <3 h	p-value no sport *vs* ≥3 h	p-value sport <3 h *vs* ≥3 h
	Mean ± SD	Mean ± SD	Mean ± SD			
Coefficient of variation (%)	36.13 ± 5.42	33.42 ± 4.96	35.52 ± 6.38	0.02	0.52	0.06
SD (mg/dl)	63.64 ± 14.04	56.84 ± 11.90	57.55 ± 13.83	0.05	0.07	0.96
HbA1c (%)	7.77 ± 1	7.59 ± 1.2	7.25 ± 0.88	0.36	**0.01**	0.17
TIR	54.76 ± 16.04	58.17 ± 20.46	64.11 ± 16.28	0.29	**0.006**	0.16
TAR	42.84 ± 17.29	39.66 ± 21.79	32.72 ± 16.35	0.35	**0.006**	0.12
TAR >250 (mg/dl)	15.31 ± 11.24	13.42 ± 12.78	9.80 ± 8.76	0.25	**0.01**	0.31
TBR	2.41 ± 2.79	2.18 ± 2.27	3.02 ± 4.31	0.66	0.43	0.24
TBR <54(mg/dl)	0.57 ± 1.11	0.32 ± 0.44	0.68 ± 1.59	0.42	0.86	0.50
Mean glucose (mg/dl)	176.30 ± 28.71	171 ± 34.33	161.4 ± 25.21	0.34	**0.01**	0.19

bold = statistically significant values.

## Discussion

In our study we analyzed the data of 202 young T1D patients in the period before and during the lockdown. All CGM parameters of glycemic control improved significantly during the lockdown period (TIR, TAR, TBR, median blood glucose and estimated HbA1c), despite the significant reduction in hours of physical activity. This improvement could be due to changes in daily rhythms caused by the closure of schools, universities, sport trainings and extra-school activities, and most likely both from greater parental controls and ease of diabetes management (waiting times between insulin administration and the start of meal, accurate carbohydrate count) (35, 36). Therefore, the negative effect due to the reduction in weekly sports activity was completely offset and overcome by the positive ”lockdown effect”, an effect in fact opposite to what was initially predicted by clinicians, but in line with the data available in the literature. Studies in the adult population showed improvement in glycemic control (19–28) unlike those on the pediatric and adolescent population which showed discordant data. Tornese et al. (29) showed glycemic improvement in a small and selected sample of patients (13 subjects, hybrid closed loop—HCL users). Brener et al. (30) showed a relatively stable glycemic control in pediatric patients with T1D and an association between a lower socioeconomic cluster and TIR in younger patients. Cristophoridis et al.** (31) demonstrated that TIR did not differ significantly before and during the lockdown period; this study was conducted on a larger number of selected patients (34 subjects, predictive low glucose suspend—PLGS users). Di Dalmazi et al. (32) showed the improvement in CGM metrics in children and adults during lockdown, whereas TIR remained unchanged in teenagers. A study conducted in India by Verma et al.** (33) showed a worsening in the glycemic trend of pediatric patients; only SMBG data monitoring were analyzed in this study and glycemic control deteriorated mainly due to unavailability of inuslin/glucostrips during the lockdown period. Another study conducted in India by Shah et al. (34) showed a stable glycemic control in children during lockdown, highlighting importance of stronger family support system leading to more steady daily routine. Therefore, we decided to collect data from a large population of children, adolescents and young adults, not overly selected for the type of therapy, and to stratify the data analysis by age groups, to clarify the effect of age on glycemic control during the lockdown.

The data show a reduction in physical activity across the study population. In Italy, the restrictions imposed by the government have greatly reduced the possibilities of participating in sports outside. Gyms, swimming pools, sports fields and playgrounds have been closed. In addition, the sports clubs have suspended activities and training. The only sports allowed were in-home activity or running alone in the neighborhood. There were no major changes in climatic conditions between T0 and T1 (+4°C in mean temperature, and +3 rainy days between T0 and T1). The stratified analysis of data by age group raises some interesting considerations. In the group of “primary school children” a reduction in sports activity was observed during the lockdown. Despite the small number of patients in this group and the unclear reasons for such reduction, it is reasonable to believe that it was not possible for the parents to have their children carry out the physical activity that was regularly scheduled in sports center and the children did not participate consistently in online workouts. Furthermore, younger patients are already normally under strict control of adults and the chances of not respecting the behaviors for the correct management of diabetes are rare even outside lockdown conditions; this could explain the less beneficial “lockdown effect” on glycemic control in this age group than in the others.

The data show that glycemic improvement increases proportionally to the age of the patients. Daily stress has a negative impact on glycemic control and is greater in puberty, adolescence and adulthood (36) and although quarantine was a stressful psychological condition, it seems that the young patients have reacted with high levels of resilience to this situation (37). In addition, teenagers and young adults are less controlled by parents, may not follow adequate behaviors for the correct management of therapies and have a much worse dietary lifestyle (dinners out of home, aperitifs, snacks) than children. It is also interesting to point out that during the lockdown the Italian families rediscovered the pleasure of cooking simpler and healthier products at home (during the lockdown in the Italian supermarkets the stocks of yeast and flour ran out); therefore, the consumption of ready-made, processed foods and fast-foods has decreased and the whole population seems to have followed a healthier Mediterranean diet (38).

The study shows some interesting aspects on the role of physical activity on glycemic control during the lockdown. The beneficial effects of sport in young T1D patients are widely known (12), although this is not reflected in the data of this study. The sub-analysis of 129 patients included in “intense physical activity” group at T0 (≥3 h per week) showed the effect of changing sport habits during the lockdown: patients who stopped exercising at T1 did not improve their glycemic control, while those who continued to do physical activity improved TIR in proportion to the weekly sports hours practiced. The sub-analysis of 56 patients included in “intense physical activity” group both at T0 and at T1 shows a significant improvement in glycemic control during the lockdown (+7.2% points of TIR). Therefore, the beneficial ”lockdown effect” appears to be completely lost in patients who have adopted a sedentary lifestyle during the lockdown, while it is extremely enhanced in patients who have continued to exercise.

The role of technology in diabetes management is well known in our Regional Pediatric Diabetes Center and more than the 75% of our patients were already CGM users before the lockdown. In contrary to what was hypothesized in some other studies with a much smaller number of patients, the role of telemedicine does not seem relevant in obtaining better glycemic control during lockdown in T1D young patients (39–42). Our experience shows that the use of CGM and telemedicine has certainly played an important role for diabetes teams in maintaining contact with their patients and in continuing to support them in managing the disease, despite the fact that the number of visits does not correlate with better glycemic control. Our data allow us to conclude that glycemic improvement is not due to the increased support of the diabetes staff; in fact, the patients who required more telemedicine visits were those characterized by a worse metabolic control and who therefore needed more support.

Several studies have shown the role of socio-economic status in glycemic control during lockdown, highlighting the correlation of socio-economic deprivation and reduction of time in range in adults and the correlation between age, socio-economic status and glycemic control in children with T1D (30). In this study we did not collect data on the socio-economic status of patients and their families. Our clinical experience has shown greater difficulty in maintaining good glycemic control during lockdown in patients with lower socioeconomic status. In this complicated period, the role of technology in managing diabetes has become very important and this has prompted our center to develop technological tools more accessible to all families. To optimize the use of CGM and guarantee everyone access to the television service, we recently launched, in collaboration with our patient associations, a project to distribute smartphones to families who did not have the possibilities to acquire them.

It is well known that prolonged lockdown periods resulted in weight gain in children and adults. This issue was also evaluated in T1D patients during lockdown periods. Several studies showed weight gain during the lockdown. However, the weight gain did not change the glycemic control in the patients (43). We decided not to collect the data relating to the weight of the patients because the method of data collection (telemedicine, referenced data) did not seem sufficiently reliable for a statistical analysis.

The limitations of this study are the single-center nature and the very small sample size of “primary school children” group. However, this study is the first report that analyzed data from a large heterogeneous and not over-selected population of pediatric and young T1D patients, with a focus on the role of many factors to consider in the management of diabetes. We believe that our data and considerations may be useful to health care professionals caring for children and adolescents during the COVID-19 pandemics. The study clearly shows the aspects on which specialists should work more with patients in the unfortunate case we there will be a second lockdown: cooking at home, following a healthier and more balanced diet and not interrupting scheduled physical activity, and being ready for a physical activity tailored to the patient’s age and personal needs. Moreover, why not think about how to work better on these aspects independently of the lockdown?

In conclusion, this study showed that the lockdown led to an overall improvement in glycemic control of young T1D patients. The healthier lifestyle and the potential reduction of stress contributed to the completely unexpected “lockdown effect”, which resulted in a significant improvement in glycemic control and diabetes management.

## Data Availability Statement

The original contributions presented in the study are included in the article/supplementary material. Further inquiries can be directed to the corresponding author.

## Author Contributions

NM designed the study and wrote the manuscript. MB designed the study and wrote the manuscript. CMo researched data and reviewed the manuscript. FV researched data. CMe researched data. FP researched data. MCab researched data. MCal did statistical analysis. Gd’A reviewed the manuscript and contributed to the discussion. MM reviewed the manuscript and contributed to the discussion. All authors contributed to the article and approved the submitted version.

## Conflict of Interest

The authors declare that the research was conducted in the absence of any commercial or financial relationships that could be construed as a potential conflict of interest.

## References

[1] Del Rio C Malani PN. COVID-19 New insights on a rapidly changing epidemic. JAMA (2020) 323:14:1339–40. 10.1001/jama.2020.3072 32108857

[2] Wu C Chen X Cai Y Xia J Zhou X Xu S. Risk Factors Associated With Acute Respiratory Distress Syndrome and Death in Patients With Coronavirus Disease 2019 Pneumonia in Wuhan, China. JAMA Intern Med (2020) 180(7):934–43. 10.1001/jamainternmed.2020.0994 PMC7070509 32167524

[3] Huang C Wang Y Li X Ren L Zhao J Hu Y. Clinical Features of Patients Infected With 2019 Novel Coronavirus in Wuhan, China. Lancet (2020) 395(10223):497–506. 10.1016/S0140-6736(20)30183-5 31986264 PMC7159299

[4] CDC COVID-19 Response Team. Preliminary Estimates of the Prevalence of Selected Underlying Health Conditions Among Patients With Coronavirus Disease 2019 – United States, February 12–March 28. MMWR Morb Mortal Wkly Rep (2020) 69:382–6. 10.15585/mmwr.mm6913e2 PMC7119513 32240123

[5] Shenoy A Ismaily M Bajaj M. Diabetes and COVID-19: A Global Health Challenge. BMJ Open Diabetes Res Care (2020) 8(1):e001450. 10.1136/bmjdrc-2020-001450 PMC7222578 32345580

[6] Guo W Li M Dong Y Zhou H Zhang Z Tian C. Diabetes Is a Risk Factor for the Progression and Prognosis of COVID-19. Diabetes Metab Res Rev (2020) 31:e3319. 10.1002/dmrr.3319 PMC7228407 32233013

[7] CDC COVID-19 Response Team. Coronavirus Disease 2019 in Children – United States, February 12–April 2, 2020. MMWR Morb Mortal Wkly Rep (2020) 69:422–6. 10.15585/mmwr.mm6914e4 PMC7147903 32271728

[8] Ebekozien OA Noor N Gallagher MP Alonso GT. Type 1 Diabetes and COVID-19: Preliminary Findings From a Multicenter Surveillance Study in the U.S. Diabetes Care (2020) 43(8):e83–5. 10.2337/dc20-1088 PMC7372041 32503837

[9] Jiatong S Wenjun L. Epidemiological Characteristics and Prevention and Control Measures of Corona Virus Disease 2019 in Children. J Trop Med (2020) 20(2):153–6.

[10] Lee PI Hu YL Chen PY Huang YC Hsueh PR. Are Children Less Susceptible to COVID-19? J Microbiol Immunol Infect (2020) 53(3):371–2. 10.1016/j.jmii.2020.02.011 PMC7102573 32147409

[11] Ludvigsson JF. Systematic Review of Covid-19 in Children Shows Milder Cases and a Better Prognosis Than Adults. Acta Paediatr (2020) 109(6):1088–95. 10.1111/apa.15270 PMC7228328 32202343

[12] Riddell MC Gallen IW Smart CE Taplin CE Adolfsson P Lumb AN. Exercise Management in Type 1 Diabetes: A Consensus Statement. Lancet Diabetes Endocrinol (2017) 5(5):377–90. 10.1016/S2213-8587(17)30014-1 28126459

[13] Lee SWH Ooi L Lay YK. Telemedicine for the Management of Glycemic Control and Clinical Outcomes of Type 1 Diabetes Mellitus: A Systematic Review and Meta-Analysis of Randomized Controlled Studies. Front Pharmacol (2017) 8:330. 10.3389/fphar.2017.00330 28611672 PMC5447671

[14] Szypowska A Ramotowska A Dzygalo K Golicki D. Beneficial Effect of Real-Time Continuous Glucose Monitoring System on Glycemic Control in Type 1 Diabetic Patients: Systematic Review and Meta-Analysis of Randomized Trials. Eur J Endocrinol (2012) 166(4):567–74. 10.1530/EJE-11-0642 22096111

[15] Slover RH 2nd. Continuous Glucose Monitoring in Children and Adolescents. Curr Diab Rep (2012) 12(5):510–6. 10.1007/s11892-012-0303-6 22791108

[16] Battelino T Conget I Olsen B Schütz-Fuhrmann I Hommel E Hoogma R. The Use and Efficacy of Continuous Glucose Monitoring in Type 1 Diabetes Treated With Insulin Pump Therapy: A Randomised Controlled Trial. Diabetologia (2012) 55(12):3155–62. 10.1007/s00125-012-2708-9 PMC3483098 22965294

[17] Beck RW Riddlesworth T Ruedy K Ahmann A Bergenstal R Haller S. Effect of Continuous Glucose Monitoring on Glycemic Control in Adults With Type 1 Diabetes Using Insulin Injections: The DIAMOND Randomized Clinical Trial. JAMA (2017) 317(4):371–8. 10.1001/jama.2016.19975 28118453

[18] JDRF CGM Study Group. JDRF Randomized Clinical Trial to Assess the Efficacy of Real-Time Continuous Glucose Monitoring in the Management of Type 1 Diabetes: Research Design and Methods. Diabetes TechnolTher (2008) 10(4):310–21. 10.1089/dia.2007.0302 18828243

[19] Bonora BM Boscari F Avogaro A Bruttomesso D Fadini GP. Glycaemic Control Among People With Type 1 Diabetes During Lockdown for the SARS-CoV-2 Outbreak in Italy. Diabetes Ther (2020) 11(6):1–11. 10.1007/s13300-020-00829-7 PMC7213551 32395187

[20] Fernandez E Cortazar A Bellido V. Impact of COVID-19 Lockdown on Glycemic Control in Patients With Type 1 Diabetes. Diabetes Res Clin Pract (2020) 166:108348. 10.1016/j.diabres.2020.108348 32711000 PMC7375311

[21] Mesa A Viñals C Pueyo I1 Roca D Vidal M Giménez M. The Impact of Strict COVID-19 Lockdown in Spain on Glycemic Profiles in Patients With Type 1 Diabetes Prone to Hypoglycemia Using Standalone Continuous Glucose Monitoring. Diabetes Res Clin Pract (2020) 167:108354. 10.1016/j.diabres.2020.108354 32739380 PMC7392049

[22] Dover AR Ritchie SA McKnight JA Strachan MWJ Zammitt NN Wake DJ. Assessment of the Effect of the COVID-19 Lockdown on Glycaemic Control in People With Type 1 Diabetes Using Flash Glucose Monitoring. Diabetes Med (2021) 38(1):e14374. 10.1111/dme.14374 PMC7436620 32740984

[23] Capaldo B Annuzzi G Creanza A Giglio C De Angelis R Lupoli R. Blood Glucose Control During Lockdown for COVID-19: CGM Metrics in Italian Adults With Type 1 Diabetes. Diabetes Care (2020) 43(8):e88–9. 10.2337/dc20-1127 PMC7372051 32540921

[24] Longo M Caruso P Petrizzo M Castaldo F Sarnataro A Gicchino M. Glycemic Control in People With Type 1 Diabetes Using a Hybrid Closed Loop System and Followed by Telemedicine During the COVID-19 Pandemic in Italy. Diabetes Res Clin Pract (2020) 169:108440. 10.1016/j.diabres.2020.108440 32926958 PMC7486201

[25] Aragona M Rodia C Bertolotto A Campi F Coppelli A Giannarelli R. Type 1 Diabetes and COVID-19: The “Lockdown Effect”. Diabetes Res Clin Pract (2020) 170:108468. 10.1016/j.diabres.2020.108468 32987040 PMC7518840

[26] Moreno-Domínguez Ó González-Pérez de Villar N Barquiel B Hillman-Gadea N Gaspar-Lafuente R Arévalo-Gómez M. Factors Related to Improvement of Glycemic Control Among Adults With Type 1 Diabetes During Lockdown Due to COVID-19. Diabetes Technol Ther (2020) 23(5):399–400. 10.1089/dia.2020.0550 33237817

[27] Pla B Arranz A Knott C Sampedro M Jiménez S Hernando I. Impact of COVID-19 Lockdown on Glycemic Control in Adults With Type 1 Diabetes Mellitus. J Endocr Soc (2020) 4(12):bvaa149. 10.1210/jendso/bvaa149 33173841 PMC7641317

[28] Potier L Hansel B Larger E Gautier JF Carreira D Assemien R. Stay-At-Home Orders During the COVID-19 Pandemic, an Opportunity to Improve Glucose Control Through Behavioral Changes in Type 1 Diabetes. Diabetes Care (2021) 44(3):839–43. 10.2337/dc20-2019 33361146

[29] Tornese G Ceconi V Monasta L Carletti C Faleschini E Barbi E. Glycemic Control in Type 1 Diabetes Mellitus During COVID-19 Quarantine and the Role of In-Home Physical Activity. Diabetes Technol Ther (2020) 22(6):462–7. 10.1089/dia.2020.0169 32421355

[30] Brener A Mazor-Aronovitch K Rachmiel M Levek N Barash G Pinhas-Hamiel O. Lessons Learned From the Continuous Glucose Monitoring Metrics in Pediatric Patients With Type 1 Diabetes Under COVID-19 Lockdown. Acta Diabetol (2020) 57(12):1511–7. 10.1007/s00592-020-01596-4 PMC7538839 33026497

[31] Christoforidis A Kavoura E Nemtsa A Pappa K Dimitriadou M. Coronavirus Lockdown Effect on Type 1 Diabetes Management on Children Wearing Insulin Pump Equipped With Continuous Glucose Monitoring System. Diabetes Res Clin Pract (2020) 166:108307. 10.1016/j.diabres.2020.108307 32650036 PMC7340587

[32] Di Dalmazi G Maltoni G Bongiorno C Tucci L Di Natale V Moscatiello S. Comparison of the Effects of Lockdown Due to COVID-19 on Glucose Patterns Among Children, Adolescents, and Adults With Type 1 Diabetes: CGM Study. BMJ Open Diabetes Res Care (2020) 8(2):e001664. 10.1136/bmjdrc-2020-001664 PMC7594202 33115820

[33] Verma A Rajput R Verma S Balania VKB Jangra B. Impact of Lockdown in COVID 19 on Glycemic Control in Patients With Type 1 Diabetes Mellitus. Diabetes Metab Syndr (2020) 14(5):1213–6. 10.1016/j.dsx.2020.07.016 PMC7357511 32679527

[34] Shah N Karguppikar M Bhor S Ladkat D Khadilkar V Khadilkar A. Impact of Lockdown for COVID-19 Pandemic in Indian Children and Youth With Type 1 Diabetes From Different Socio-Economic Classes. J Pediatr Endocrinol Metab (2020) Nov 13 34(2):217–23. 10.1515/jpem-2020-0460 33185578

[35] Hall G Laddu DR Phillips SA Lavie CJ Arena R. A Tale of Two Pandemics: How Will COVID-19 and Global Trends in Physical Inactivity and Sedentary Behavior Affect One Another? Prog Cardiovasc Dis (2020) S0033-0620(20):30077–3. 10.1016/j.pcad.2020.04.005 PMC7194897 32277997

[36] Lukács A Mayer K Sasvári P Barkai L. Health-Related Quality of Life of Adolescents With Type 1 Diabetes in the Context of Resilience. Pediatr Diabetes (2018) 19(8):1481–6. 10.1111/pedi.12769 30203556

[37] Passanisi S Pecoraro M Pira F Alibrandi A Donia V Lonia P. Quarantine Due to the COVID-19 Pandemic From the Perspective of Pediatric Patients With Type 1 Diabetes: A Web-Based Survey. Front Pediatr (2020) 8:491. 10.3389/fped.2020.00491 32850562 PMC7411347

[38] Mańkiewicz-Żurawska I Jarosz-Chobot P. Nutrition of Children and Adolescents With Type 1 Diabetes in the Recommendations of the Mediterranean Diet. Pediatr Endocrinol Diabetes Metab (2019) 25(2):74–80. 10.5114/pedm.2019.85817 31343138

[39] Nørgaard C. Telemedicine Consultations and Diabetes Technology During COVID-19. J Diabetes Sci Technol (2020). 10.1177/1932296820929378 PMC7673191 32429702

[40] Peters AL Garg SK. The Silver Lining to COVID-19: Avoiding Diabetic Ketoacidosis Admissions With Telehealth. Diab Technol Ther (2020) 22(6):449–53. 10.1089/dia.2020.0187 32383989

[41] Predieri B Leo F Candia F Lucaccioni L Madeo SF Pugliese M. Glycemic Control Improvement in Italian Children and Adolescents With Type 1 Diabetes Followed Through Telemedicine During Lockdown Due to the COVID-19 Pandemic. Front Endocrinol (Lausanne) (2020) 11:595735. 10.3389/fendo.2020.595735 33424771 PMC7793913

[42] Parise M Tartaglione L Cutruzzolà A Maiorino MI Esposito K Pitocco D. Teleassistance for Patients With Type 1 Diabetes During the COVID-19 Pandemic: Results of a Pilot Study. J Med Internet Res (2021) 23(4):e24552. 10.2196/24552 33769945 PMC8025914

[43] Ruissen MM Regeer H Landstra CP Schroijen M Jazet I Nijhoff MF. Increased Stress, Weight Gain and Less Exercise in Relation to Glycemic Control in People With Type 1 and Type 2 Diabetes During the COVID-19 Pandemic. BMJ Open Diabetes Res Care (2021) 9(1):e002035. 10.1136/bmjdrc-2020-002035 PMC7802391 33431602

